# ParkProTrain: an individualized, tablet-based physiotherapy training programme aimed at improving quality of life and participation restrictions in PD patients – a study protocol for a quasi-randomized, longitudinal and sequential multi-method study

**DOI:** 10.1186/s12883-019-1355-x

**Published:** 2019-06-25

**Authors:** Carolin Siegert, Björn Hauptmann, Nicole Jochems, Andreas Schrader, Ruth Deck

**Affiliations:** 1 Department Rehabilitation Sciences, Institute for Social Medicine and Epidemiology, University of Lübeck, Ratzeburger Allee 160, 23562 Lübeck, Germany; 2 Segeberger Kliniken GmbH, Neurological Centre, Bad Segeberg, Germany; 3 Department Performance, Neuroscience, Therapy and Health, Medical School Hamburg, Hamburg, Germany; 4 Institute for Multimedia and Interactive Systems, University of Lübeck, Lübeck, Germany; 5 Institute of Telematics, University of Lübeck, Lübeck, Germany; 6 Institute for Social Medicine and Epidemiology, University of Lübeck, Lübeck, Germany

**Keywords:** Parkinson’s disease, Quality of life, Participation, Exercise, Physiotherapy, Telemedicine, App, Tablet

## Abstract

**Background:**

Parkinson’s disease (PD) is one of the most common neurodegenerative diseases. Patients suffer from a variety of motor and non-motor symptoms that severely affect their daily lives and quality of life. In many cases, a three-week inpatient Parkinson’s complex treatment (MKP) can improve the overall condition and quality of life of patients in a short time. In the outpatient sector, however, there is often a lack of human resources and structures necessary for the interdisciplinary treatment of the disease. To support PD patients in continuing the physical exercises they learned from the MKP on a regular basis, a tablet-based training programme will be developed in which exercises can be adjusted to the patient’s abilities. This programme is expected to increase quality of life and social participation, as well as delay the progression of the impairment.

**Methods:**

a) Quasi-randomized, prospective longitudinal study (sequential study design). The intervention group receives a tablet-based training programme during and for 9 months after the MKP, and the control group receives treatment as usual. The evaluation is carried out by means of a written survey at three points in time (the beginning and end of the MKP and after 9 months). b) Qualitative analysis of interviews and focus groups in terms of feasibility and acceptance. c) Formative evaluation of the app and the administration panel. d) Evaluation of the implementation of the training programme by analysing the planned and performed physical activities, as well as evaluation of the phone calls between physiotherapists and patients.

**Discussion:**

The tablet-based training programme can ensure continuous and long-term support for PD patients. They learn different self-management strategies during and after their MKP and are empowered to assume responsibility for carrying out regular physical activity on their own. Because common app stores have no scientifically evaluated apps for PD patients in the German language, the app can fill this gap and help PD patients receive high-quality care in the implementation of physically activating exercises regardless of their place of residence. In addition, the user-centred development of the app ensures that the app meets the specific needs of PD patients.

**Trial registration:**

German Register of Clinical Trials, drks.de. Identifier: DRKS00014952. Registered on June 20th 2018. Date and version identifier April 25th 2019; version 1.

## Background

Parkinson’s Disease (PD) is one of the most common neurodegenerative diseases [1] with an onset usually at 65 to 70 years of age [2]. An increase in the number of affected people is expected [3]. In addition to the serious physical and mental suffering of those patients, the disease represents a heavy financial burden on the German health care system [4], and the total annual costs in Germany are currently approximately 2.8 billion euros [5]. Of this, 70% is attributable to direct medical expenses, which consist of medication costs and inpatient treatment [4]. The number of patients treated with a Parkinson’s complex treatment (Multimodale Komplexbehandlung bei Morbus Parkinson, MKP) according to OPS 8-97d has risen sharply in the last five years [6]. Further costs arise from occupational and physiotherapeutic treatments, among others [7]. Physical activity is of considerable importance to disease progression. Training practices such as physiotherapy and various physical activities (including Nordic walking, Tai Chi, dancing, strength training, treadmill training) have a positive impact on gait, endurance, strength, balance, and quality of life for patients [8–12]. The S3 guideline “Idiopathic Parkinson’s Syndrome” recommends physiotherapy, including other physical activities, as a flexible and long-term treatment strategy. A basic principle is to motivate patients to participate in regular, adaptive physical activity [13]. This is made more difficult not only by motor restrictions (rigor, akinesia) but also by non-motor symptoms. Anhedonia (joylessness), apathy, and possibly depression symptoms often present in many patients are factors that hinder an active lifestyle. The inpatient MKP includes both drug therapy and intensified activating therapy that includes physiotherapy and occupational therapy, among other treatments. MKP improves the overall condition and quality of life of PD patients in many cases [14]. After discharge, however, there is often a lack of the human resources and structures in the outpatient sector that are needed for the necessary interprofessional treatment of the disease [14]. There are almost no physiotherapists with a main focus on PD. Therefore, patients rarely receive PD-specific outpatient physiotherapy care following MKP that builds on the physically activating exercises and on the achieved results of MKP. The aim of this intervention is to achieve largely autonomous initiation and maintenance of physical activity in PD patients.

### Aims

Against this background, patients will receive a tablet-based training programme that is to be used during and after MKP (OPS: 8-97d). The training programme will help PD patients continue the physically activating physiotherapy and other physical activities (such as Tai Chi, Nordic walking, and dancing) that they learned during their MKP. The project has three goals: (1) user-centred development and implementation of an individualized tablet-based training programme, (2) transfer of the physically activating exercises learned in the MKP and other physical activities into everyday life, and (3) improvement in the quality of life and social participation of PD patients and delayed progression of impairment through the regular implementation of the tablet-based training programme.

### Intervention

The planned training programme is based on the HAPA model [15], which focuses in particular on the factors of intent formation and concrete implementation of the established health behaviour. In practice, the patient-centred 5-A-model (assess, advise, agree, assist, arrange) will also be used as an evidence-based framework for health behaviour interventions [16] to increase patients’ self-management skills and support behavioural change [17]. The training programme for PD patients is developed as an application (app). This app will be installed on a tablet, which will be given to the patients of the IG at the beginning of the MKP. The app contains videos with verbal instructions and explanations for all the physically activating exercises taught in the MKP. These exercises are available in different degrees of difficulty and promote endurance, strength and balance. Other physical activities, such as Nordic walking, Tai Chi or dancing, are also suggested. When the patient activates a specific exercise or physical activity, they may also enter personal notes.

During MKP, after completing an exercise, patients record the repetition rate or duration of the exercises on the tablet and rate them with regard to well-being, effort and fun. During the MKP, patients also take part in two patient-centred seminars. The first is primarily for acquiring knowledge and motivation. The focus of the second seminar is on preparing for everyday life at home. Each patient considers personal barriers that may arise in daily routine and strategies to overcome those barriers. Possible action alternatives are defined and entered into the app. At an individual appointment at the end of the MKP, patients plan their personal home training programme on the tablet, with the support of the physiotherapist. The patient chooses exercises that match their abilities, needs and preferences. The physiotherapist assists the patient in putting together their individual training programme: she gives advice regarding the selection of the activating exercises, the degree of difficulty and the number of repetitions. The exercises and the adaptation of the exercises over the weeks are based on the learning method of behaviour shaping in operant conditioning. Here, the desired motor behavioural goal is achieved by gradually increasing effort and difficulty, practising repetitive exercises and constantly adjusting the exercises to the patient’s level of ability. Shaping has proven to be an effective training method in neurological movement disorders [18]. Patients will set a date and time for at least three 30-min sessions of physiotherapy exercises and for at least one physical activity per week for the next three weeks at home. The app displays this individually scheduled training programme in a calendar function. At home, the app reminds patients through a sound signal and/or push notification to execute their exercise programme at the scheduled time. Postponing a session (e.g., due to temporary aggravation of motor impairments, so-called off periods) is possible. The videos offer visual support in the execution of the selected exercises. After completing a scheduled training session, patients will document whether the given repetition number or duration was achieved and whether there were problems performing the exercises, and they also rate the activity in terms of well-being, effort and fun. After the end of the MKP, the data are transferred automatically from the app into an administration panel called “Cockpit” at the clinic. There, the supervising physiotherapist can monitor adherence by controlling the patient’s documentation regarding the execution of the exercises and activities, the repetition number, incomplete video viewings, etc. To permit this, the app transmits the gathered statistics for the activities performed by the patients at regular intervals. To be independent of wireless Internet access, transmission takes place via the mobile network. Based on these data, the physiotherapist provides feedback by phone and adjusts the training programme at regular intervals after the end of the MKP. Because PD patients are prone to small movement amplitudes during exercise that they cannot perceive and therefore cannot correct by themselves, regular correction by experts is required. For this reason, in addition to the telephone contacts, the patients visit the clinic four times after their MKP. Travel expenses to the clinic will be financially reimbursed. If patients have any further need for assistance between those pre-defined appointments, they can contact their physiotherapist at the hospital. Regular, attentive care and feedback from the physiotherapist promote long-term adherence [19].

### App, tablet, administration panel

To ensure the usability and acceptance of the tablet-based training programme and of the administration panel (cockpit), the later users of the system (PD patients, physiotherapists) will be included in the entire development process (User Centred Design, DIN EN ISO 9241-210 [20]). Particular attention will be paid to the skills and abilities of PD patients and to their involvement in the design of the human-technology interface. It was shown in our own studies that interaction with a touchscreen by users with tremor could be significantly improved by appropriate adaptation of the human-technology interface [21, 22]. As part of the user-centred design approach, the design process is initially planned for the first phase, and the usage requirements are specified empirically. In the main phase, design solutions are developed to fulfil these usage requirements. These include (1) the design of the human-technology interface of the tablet-based training programme, (2) the recording and editing of PD-specific support videos for the tablet-based training programme, (3) the design and implementation of the administration panel (cockpit) for the responsible physiotherapist, and (4) the development of telematics connections between the tablets and cockpit, including encrypted transmission and persistent data storage on the clinical server. The solutions will be tested in an iterative formative evaluation and revised if necessary, followed by a final evaluation in the laboratory and possible optimization of the system.

## Methods

### Eligibility criteria

All patients admitted to our partner clinic for a three-week MKP with a diagnosis of idiopathic Parkinson’s syndrome will be eligible to participate (ICD G20.0 and G20.1). Concomitant care and interventions as e.g. privately organized physiotherapy sessions after the MKP are allowed.

Exclusion criteria: Montreal Cognitive Assessment (MoCA) < 18 points, Berg Balance Scale < 41 points, a major depressive episode, moderate to severe dementia, and cardiovascular and orthopaedic/surgical reasons that argue against independent home training. Participants will be also excluded from the study if they are being admitted to an acute hospital during their MKP.

### Study design

A mixed-methods study [23] with different methodological components will be carried out (sections A-D). The study will be led by the Institute for Social Medicine and Epidemiology of the University of Lübeck. Recruitment will take place at a community clinic in Germany (Neurological Centre, Segeberger Kliniken GmbH in Bad Segeberg). A physiotherapist at this clinic will be in charge of screening the patients for their eligibility within the first three days of their MKP and recruiting them into the study by personally addressing potential participants, giving out the information sheet and collecting the informed consent.

### A: quantitative measures

To examine the long-term effects, we will conduct a monocentric, quasi-randomized longitudinal study with three measurement times: at the beginning (t_0_) and end of the MKP (t_1_) and 9 months after the MKP (t_2_). Recruitment of control group (CG) and intervention group (IG) will be performed in a sequential order, i.e., initially, all patients that are being admitted to our partner clinic during the first recruitment period will be consecutively assigned to the CG (treatment as usual) until the desired number of cases has been reached. The second recruitment period starts after the recruitment of the CG is completed. Patients that are subsequently arriving at the partner clinic for their MKP will be consecutively assigned to the IG until the desired number of cases has been reached. Neither participants nor the study personnel or care providers at the clinic will be blinded. To control the equal treatment of IG and CG during the MKP, all treatment measures that the groups receive will be continuously documented by means of an EDP-based treatment plan. All patients will complete a written questionnaire using standardized, validated instruments. Table 1 shows the core set of instruments to be used in the study.

**Table 1:** Core set of instruments used in the quantitative part of the study

Dimensions	Instruments	t_0_	t_1_	t_2_
Primary Outcome				
Quality of Life	PDQ-8 [24]	●	●	●
Secondary Outcomes				
Participation Restrictions	IMET [24]	●		●
Fear of Falling	FES-I [25]	●	●	●
Sleep Disorder	PDSS-2 [29]	●	●	●
Anxiety/Depression	PHQ-4 [31]	●	●	●
Comorbidity	SCQ-D [26]	●		●
Pain	Single Items [27]		●	
Performance Capability	Single Items [28]	●		●
Physical Activity	Federal Health Survey [29]	●		●
Moderating Variables				
Body Height, Weight	Single Items	●		●
Use of Health Services (Medical and Therapeutic Treatments, Hospital Stays, Medication, etc.)	Single Items	●		●
Sociodemographic Data	Single Items [30]	●		●

t_**0**_ = baseline/right before MKP; t_**1**_ = 3-week follow-up/right after MKP; t_**2**_ = 9 months after t_**1**_

#### Primary outcome

PD-specific quality of life will be recorded using the PDQ-8 (Parkinson’s Disease Questionnaire, German version). It contains 8 questions rated on a five-point scale assessing aspects of mobility, everyday activities, emotional well-being, stigma, social support, cognition, communication, and physical discomfort. High values indicate low quality of life. It is a valid and reliable instrument for measuring the quality of life in people with PD [24].

#### Secondary outcomes

*Participation restrictions* will be recorded with the IMET. This ICF-oriented [25] instrument determines the subjective impairment of chronically ill people in everyday life. It surveys restrictions on participation in nine everyday areas using a scale from 0 to 10 and can be evaluated both at the individual item level and as a total score. High values indicate high participation restrictions. The IMET has proven valid and reliable for use in chronically ill patients [26, 27].

*Falling anxiety* will be measured with the German version of the Falls Efficacy Scale International Version (FES-I). The questionnaire consists of 16 items rated on a four-point scale, which are combined into a total score. High values indicate high fear of falling. Several studies have reported this instrument’s high reliability and validity [28].

*Sleep problems* will be measured with the German version of the Parkinson’s Disease Sleep Scale (PDSS-2). The scale contains 15 questions rated on a five-point scale that address PD-specific sleep disorders. The items are summarized in a sum scale. High values indicate a high level of sleep disturbances. Reliability and validity can be considered good [29].

To assess *anxiety and depression*, the short version of the Health Questionnaire for Patients (PHQ-4) will be used. The questionnaire captures anxiety and depression with two questions each that are rated on a four-point scale. Sum scores are calculated, and higher values imply higher levels of anxiety and/or depression. Reliability and validity are considered good [30, 31].

*Comorbidity* will be measured by a modified version of the comorbidity questionnaire of Sangha [32], which includes total of 14 diseases that are recorded dichotomously.

The severity of *pain* in different parts of the body will be assessed on a five-point scale [33].

*Performance capability* in the domains of work, everyday life and leisure is quantified using numerical scales from 0 to 10, with high values indicating high performance. The three items have been shown in routine surveys to be a valid method for determining performance limitations [34, 35]. *Physical activity* is measured using the instrument used by Mensink in the 1999 Federal Health Survey [36]. *Sociodemographic data* will be assessed using single items [37].

### B: qualitative measures

#### Interviews and focus groups with patients, therapists and doctors

Patient experiences with the tablet-based training programme will be assessed in four focus groups of PD patients at the end of the MKP. The particular focus will be on the personal use and manageability of the app, the satisfaction with the patient-centred seminars and the individually created training plan. In addition, qualitative individual interviews with 16 patients after MKP will be carried out. The interviews will take place twice: The first time at week 9 and the second time at week 36 after the MKP, when the patients will come to the clinic for their onsite check-up. They will focus on the feasibility of implementing the individualized training plan at home; on satisfaction with the app, the phone calls and the personal appointments in the clinic; and on the adjustments to the training programme. In addition, the wishes and needs of the patients with regard to further development or optimization of the training programme and app will be recorded. To achieve a sample composition that is as heterogeneous as possible [38], patients of different genders and with different degrees of disease severity, education and extent of physical activity will be recruited prior to the MKP for the interviews. The perspective of doctors and therapists will be taken into account by means of a focus group with the specialist staff of the clinic. This focus group will take place at the end of the recruitment process in the clinic and will address the assessment of the patients’ use of the tablet-based training programme during inpatient treatment from a medical and therapeutic point of view. In particular, the practicality of the app for the patients and an assessment of the extent to which the app supports the therapeutic and medical work during the MKP and the preparation for the time at home will be in the foreground. In addition, individual interviews will be conducted after the MKP with the physiotherapist responsible for PD patients. These interviews will concern the practicality of evaluating the data with the help of the cockpit, the telephone calls and the feasibility of adapting the training programmes. All interviews and focus groups will be conducted on a guided basis. Both the guidelines and the results of the interviews will be presented and discussed among the project team and in an interdisciplinary expert forum (“Qualitative Methods Working Group”, AQUAM). The interviews will take approximately 30 min, and the focus groups will take longer. After the participants have given their consent, all interviews and focus groups will be recorded digitally and transcribed literally. The evaluation will be based on the method of qualitative content analysis [39, 40].

### C: formative evaluation of the tablet-based training programme and the administration panel (cockpit)

The formative evaluation will examine the usability of the app and the cockpit during app development. The evaluation will take place at the Usability Laboratory of the Institute for Multimedia and Interactive Systems. For this purpose, prototypes of the app and the cockpit will be developed, and potential users (PD patients) will be asked to perform sample tasks, such as playing a training video, with the prototypes. To evaluate the app, methods that gather both objective data (processing time and accuracy, human error, mental load, finding weak points) and subjective data on satisfaction and acceptance (e.g., QUIS [41], UTAUT [42]) and mental stress (NASA-TLX [43]) will be combined.

Positively evaluated design aspects will flow into the development of the overall system. At the end of the intervention, the tablet-based training programme will be evaluated by the PD patients, and the cockpit will be evaluated by the physiotherapist. The evaluation of the usability of the app and the cockpit will be based on the ISONORM questionnaire [44], which covers the implementation of the dialogue criteria by means of 21 items based on the International Ergonomics Standard DIN EN ISO 9241-110. In addition to the quality characteristic of suitability for use, subjective factors of use will be taken into account. For this purpose, the AttrakDiff questionnaire [45] will be used to measure the perceived hedonic and pragmatic quality.

### D: evaluation of the training programme’s implementation

In addition to surveying practicality and satisfaction with the app (Section B), the training programme will be evaluated with regard to its content. For this, the planned and performed physical activities, the percentage of achieved repetition numbers, the adjustments to the training plan and the reasons for postponing scheduled appointments will be analysed. Additionally, the content of the telephone calls will be analysed to gain deeper insight into the specific needs of the patients.

### Data management

#### Patient documentation

For the uniform organization and documentation of the flow of subjects in the clinic, an automated Excel documentation file will be generated, and the person responsible for the study will be trained to use it before the recruitment process begins. Each patient will be assigned a four-digit identification number (ID) (pseudonymisation of the data). The first digit will encode the assignment to the IG or CG, and the subsequent digits indicate the number of recruited respondents. All personal data will be documented on a separate data sheet in an Excel file. A corresponding link will be made via the ID. Before transferring the data to the Institute for Social Medicine and Epidemiology, these personal data will be deleted. The collection of these data will take place exclusively in the clinic for standardized documentation. The collection and storage of the data from the questionnaires will be performed separately.

### Data entry and control

The entry of data from the questionnaires in the quantitative study part (A) will be carried out by a student assistant under the guidance of a scientific employee. The entered data will be checked for random double entries (5–10%) by examining their validity and plausibility.

### Data monitoring

It is a non-drug study investigating the health benefits by a training programme fitted to the individual abilities of the patients. Therefore, harmful or adverse events are not expected. Because it is a study with minimal to no risks, there is no need of a data monitoring committee. An interim analysis is not planned.

### Case number calculation / sample size

With regard to the changes in the primary outcome, we assume that both groups (the CG and IG) will have improved in a similar way at the end of the MKP. According to our assumptions, the effect of the CG during the subsequent period will approach the starting level again, while the positive changes in the IG will stabilize or even increase slightly. To estimate the required sample size, we have referred to the results of existing studies on the influence of physical activity on quality of life (PDQ-8) in PD patients. For the effect determination, the data of Ebersbach et al. [46], Morris et al. [47] and Nadeau et al. [10] are being consulted. In Ebersbach et al., the quality of life over a 4-month course showed effect sizes of average magnitude (ES = 0.47) for patients who underwent physical training (Nordic walking), whereas there was no change in the CG. Morris et al. also report a significantly improved quality of life, with a mean effect size of ES = 0.45, after muscle training over a 3-month span. In their controlled study on the effect of intensive treadmill training (speed and incline) on quality of life, Nadeau et al. found an effect size of ES = 0.73 over a 6-month course; for simple treadmill training, they found an effect strength of ES = 0.21. In the CG, the quality of life did not change. For the calculation of the number of cases, we assume that participation in the tablet-based training programme (IG) will lead to clinically relevant positive effects (t_0_ vs. t_2_ ES = 0.40) on quality of life (PDQ-8) 9 months after the end of MKP. In contrast to the IG, there will be no changes in the CG at t_2_. To demonstrate differences between the IG and CG 9 months after MKP on the order of at least ES = 0.4 with two-sided testing at α = 5% and a power of 80%, a group size of *N* = 100 net is required for each IG and CG. We expect only a moderate dropout rate of 25% among patients after MKP due to their attachment to the clinic. Thus, to be able to evaluate N = 100 patients per group, 133 participants per study group must initially be included. Approximately 120 PD patients participate in the MKP per quarter at the partner clinic. Based on the defined inclusion and exclusion criteria, we expect 70 patients per quarter to be included in the study. The recruiting of the CG and IG will take place over a period of nine months each. Taking into account a rate of study refusal of 25%, the targeted number of cases in the clinic can be recruited within this time frame.

## Statistical methods

### Quantitative analyses

Repeated analysis of variance will be used to analyse the longitudinal data, a contingency table analysis will be used for nominal and ordinal parameters, and chi-square tests and t-tests will be used for subgroup analysis. Subgroup analyses are planned for age, sex and education (boundaries: high vs. low). For the evaluations only complete cases are used. The analyses will be performed using the statistics programme SPSS 22.0.

### Qualitative analysis of the interviews and focus groups

Qualitative content analysis [39, 40, 48, 49] will be used to evaluate the interviews and focus groups. The evaluations will be carried out with the programme MAXQDA 12. To systematically describe content-relevant topics using a category system, all transcripts will be reviewed to identify interesting content-related aspects. The main topics (main categories) will be derived deductively from the research questions and interview guidelines, and subcategories will be developed inductively from the material, for example, by subsumption [40]. To test the category system, two scientists will independently test-code part of the material, followed by a modification of the categories and their definitions. The entire coding process will be performed using consensual coding [49], i.e., the transcripts will initially be coded independently by two scientists. Afterwards, the codings will be brought together, and a consensus will be reached.

### Analysis of the cockpit data

In the cockpit, the relevant user statistics will be continuously updated and visualized. The final features of the cockpit visualizations will be defined during the user-centred design process, but certain functions are canonical. Envisaged are, among other features, graphical displays showing frequencies, duration and cessations of video sequences. This information can be inspected individually for a particular patient or comparatively across all patients.

## Discussion

Regular physical activity has high curative potential for chronic and degenerative diseases [50]. Against the background of demographic change, a higher number of chronically ill and elderly people are expected to need support for carrying out physical activity. In addition, the care of PD patients in an outpatient setting is deficient (especially in rural areas). Through this proposed tablet-based programme, patient competence and compliance can be increased and the general aftercare improved. Additionally, the app can easily be customized and extended to meet other indications. It could thus be made available to all people who need help with home-based training.

### Innovation factor

For the first time, our tablet-based training programme ensures continuous and long-term support for PD patients. Physically activating exercises and physical activities are initiated at the beginning of the MKP and continued at home. PD patients learn various self-management strategies during and after their MKP and are empowered to assume responsibility for carrying out regular physical activity on their own. Through the tablet-based training programme, all PD patients receive high-quality care in the implementation of physically activating exercises and physical activity, regardless of their place of residence. Finally, our project also closes a gap at the level of available applications. In the most common app stores, there is no scientifically evaluated app in German that is integrated into the overall treatment of the patient and could support the patients in self-managing their disease. In addition, the user-centred development of the app ensures that the app meets the specific needs of PD patients.

### Immediate expected results of the project

Through the personalized training programme, we expect an increase in physical activity that positively affects the motor and non-motor impairments of PD patients and thus improves quality of life. Due to the regular supervision and adaptation of the training programme, we assume that the positive effects of MKP can be maintained longer and that a slowing down of the course of the disease can be achieved. Due to the high proportion of self-performed physically activating exercises and physical activity in our intervention, individualized, time-consuming care by a therapist can be significantly reduced. It can be assumed that costs can significantly be saved in comparison to conventional occupational and/or physiotherapy prescriptions. The physical and everyday obstacles that PD patients face in executing and maintaining physical activity are considered in our individual training plan and in the preparatory and accompanying measures (such as patient-centred seminars and telephone and in-person support). Thus, the intervention meets the specific needs and requirements of this particular patient clientele and should therefore achieve high acceptance and adherence.

### Transferability of the project results to the everyday care situation

The intervention to be examined in this study will be established and evaluated as an example in a specialty clinic for the treatment of PD. In principle, however, this intervention can also be transferred to other clinics. Patients’ desires for long-term and continuous care are met in this intervention. The exact time and personnel resources required for the implementation of the intervention (for telephone calls, onsite appointments) will be measured in the context of this project and are therefore available as key data for the establishment of the programme in other clinics.

The app to be developed in this project can be integrated into other telemedicine developments for Parkinson’s treatment [51]. The basic structure of server-side cockpit functionality, data transfer through the app, and graphical user interfaces is implemented modularly. The frontend for the selection of videos can therefore be easily replaced or supplemented by alternative modules with a different content focus.

## Limitations

A disease-specific (but rather small) risk is that the disease might deteriorate significantly. In this case, an exercise adjustment will be made in the “negative direction” to ensure that the patient does not feel overwhelmed and can continue training despite deterioration. From a technical point of view, there are risks concerning the possible failure of tablets and the possible breakdown of the data connection in the mobile radio channels. For data transmission, an intelligent mechanism is implemented that repeats transmission attempts in cases of failure after variable time intervals. Because the data are not time-critical, this problem is negligible. In the event of malfunctioning terminal devices, a pool of replacement devices and a strategy for easily transferring the patient-specific settings to the replacement device are maintained. Data security is planned on two stages. In addition to the use of conventional encryption techniques on the data lines, specific encodings of the semantic meaning of the user statistics will be developed that cannot be interpreted by outsiders. Personal data will never be transmitted via a data line. Another risk could arise if changes occur in the structural characteristics of the treated patients after recruitment of the CG is completed. To detect this risk, patient characteristics will be collected and compared for both groups. Existing differences will be adequately included in the analyses (e.g., as covariates in multivariate methods or by propensity score matching). Based on our current knowledge, we assume that the occurrence of such relevant structural changes in the context of the project is unlikely.

## Data Availability

The datasets generated and/or analysed during the current study are not publicly available. Declaration of consent does not include release of the data for external analyses. A model consent form is available from the corresponding author by reasonable request. Trial results will be published and thus be made available to anyone interested in the study. It is not planned to make IPD available. The trial protocol, participant-level dataset, and statistical code for generating the results will also not be made publicly available.

## Abbreviations

MKP: Multimodale Komplexbehandlung bei Morbus Parkinson is a three-week inpatient programme for PD patients in which they receive drug therapy as well as units in physiotherapy, occupational, music, sports and speech therapy and neuropsychology.
OPS: Operationen- und Prozedurenschlüssel (the German Operations and Procedures Key) is used to encode operations and medical procedures in inpatient care as well as in outpatient surgery in the clinic.
PD: Parkinson’s disease
