# Correction to: ParkProTrain: an individualized, tablet-based physiotherapy training programme aimed at improving quality of life and participation restrictions in PD patients–a study protocol for a quasi-randomized, longitudinal and sequential multi-method study

**DOI:** 10.1186/s12883-019-1423-2

**Published:** 2019-08-17

**Authors:** Carolin Siegert, Björn Hauptmann, Nicole Jochems, Andreas Schrader, Ruth Deck

**Affiliations:** 1 Department Rehabilitation Sciences, Institute for Social Medicine and Epidemiology, University of Lübeck, Ratzeburger Allee 160, 23562 Lübeck, Germany; 2 Segeberger Kliniken GmbH, Neurological Centre, Bad Segeberg, Germany; 3 Department Performance, Neuroscience, Therapy and Health, Medical School Hamburg, Hamburg, Germany; 4 Institute for Multimedia and Interactive Systems, University of Lübeck, Lübeck, Germany; 5 Institute of Telematics, University of Lübeck, Lübeck, Germany; 6 Institute for Social Medicine and Epidemiology, University of Lübeck, Lübeck, Germany

**Correction to: BMC Neurology (2019) 19:143**

**https://doi.org/10.1186/s12883-019-1355-x**

Following publication of the original article [1], the authors reported errors in reference citations found in Table 1. The correct version of the table is provided here.

**Table 1:** Core set of instruments used in the quantitative part of the study

Dimensions	Instruments	t_0_	t_1_	t_2_
Primary Outcome				
Quality of Life	PDQ-8 [24]	●	●	●
Secondary Outcomes				
Participation Restrictions	IMET [27]	●		●
Fear of Falling	FES-I [28]	●	●	●
Sleep Disorder	PDSS-2 [29]	●	●	●
Anxiety / Depression	PHQ-4 [30]	●	●	●
Comorbidity	SCQ-D [32]	●		●
Pain	Single Items [33]	●	●	●
Performance Capability	Single Items [35]	●		●
Physical Activity	Federal Health Survey [36]	●		●
Moderating Variables				
Body Height, Weight	Single Items	●		●
Use of Health Services (Medical and Therapeutic Treatments, Hospital Stays, Medication, etc.)	Single Items	●		●
Sociodemographic Data	Single Items [37]	●		●

t_**0**_ = baseline/right before MKP; t_**1**_ = 3-week follow-up/right after MKP; t_**2**_ = 9 months after t_**1**_

The authors regret this error.
